# Beneficial Effects of Selenium and Its Supplementation on Carcinogenesis and the Use of Nanoselenium in the Treatment of Malignant Tumors

**DOI:** 10.3390/ijms252011285

**Published:** 2024-10-20

**Authors:** Monika Maleczek, Joanna Reszeć-Giełażyn, Katarzyna Szymulewska-Konopko

**Affiliations:** Department of Medical Pathomorphology, Medical University of Białystok, 15-269 Białystok, Poland; monika.maleczek@umb.edu.pl (M.M.);

**Keywords:** selenium, nano-selenium, cancer, glutathione peroxidases, oxidative stress, carcinogenesis

## Abstract

Selenium was recognized as a non-toxic element in the second half of the 20th century. Since then, the positive impact of selenium on the functioning of the human body has been noticed. It has been shown that low levels of selenium in the body are significantly associated with a higher risk of developing cancer. Selenium acts as an antioxidant and inhibits the proliferation of cancer cells. It has been shown that selenium supplementation may contribute to reducing the risk of DNA mutations and carcinogenesis. Nanomedicine has become very helpful in both the diagnosis and treatment of cancer. Due to its anticancer properties, selenium is used in nanotechnology as selenium nanoparticles.

## 1. Introduction

From the discovery of selenium (Se) in 1817 by JJ Berzelius until 1957, it was considered a highly toxic element. In the second half of the 20th century, there was a breakthrough thanks to the discovery of biochemists Schwarz and Foltz, who observed the beneficial effects of Se on tested animals. Scientists introduced yeast rich in Se into the diet of rats, which resulted in a reduction in the level of liver necrotic bodies. This addition made it possible to maintain the proper liver function of the tested animals. The previous supply of yeast low in Se resulted in the necrosis of liver cells in the tested rats. It was concluded that Se has an effect on inhibiting the level of liver cell necrosis, and since the experiment, the positive effect of Se on the body has begun to be appreciated [1,2].

The scope of Se’s action is wide: it affects, among others, cell growth and gene expression by activating transcription factors [3,4], it participates in the protection of cells against reactive oxygen species (ROS) [5,6], and in the detoxification of the body from heavy metals [5,7], regulation of the reproductive system [8,9] and immune system [10], regulation of the functioning of the thyroid gland, and has anti-inflammatory properties [11]. It is believed that it may play a significant anti-cancer role in solid-tumor cancers [12], anti-aging, and anti-neurodegenerative effects [13]. In human the body, selenium intervenes in glutathione peroxidase (GPX), iodothyronine deiodinase, and thioredoxin reductase [11]. Se, due to its unique properties, is essential for maintaining human health. Even though it occurs in small amounts in the human body, its deficiency may cause dysfunction in various systems. The physiological function of Se comes mainly from selenoproteins, which protect against oxidative stress and adverse conditions. A required supply of Se and its supplementation may have a beneficial effect on brain function, cardiovascular diseases, as well as cancer and the diseases caused by heavy metals. Cell function is regulated by nutrients and metabolites. Se has several functions and affects cell growth and development by maintaining the redox balance. The role of Se in cell death is not fully explained, but its participation is distinguished in various types of cell death: apoptosis, ferroptosis, autophagy, and pyroptosis [11].

Se, as a microelement necessary for the proper development and growth of the body, may also affect the risk of malignant tumors [12]. It has been shown using epidemiological and laboratory studies that low Se levels in the body are significantly associated with a higher risk of developing cancer of the colon, lung, and prostate [13]. In the publications available so far, two modes of anticancer action are particularly distinguished. The first is the role of Se as an antioxidant—it helps protect the body against free radicals. The protective role of Se against pro-oxidants results primarily from the presence of this element in the active center of antioxidant enzymes. The best-known protein is the enzyme glutathione peroxidase (GPX), whose leading function is to protect cellular components (lipids, DNA) against the harmful effects of hydrogen peroxide and various organic peroxides produced during metabolism in the body [12,14]. Se-dependent glutathione peroxidase (Se GPX) is more active at lower concentrations of hydrogen peroxide, while, next to Se GPX, also found in cells, non-Se-dependent glutathione peroxidase (non-Se-GPX) reduces lipid peroxides [15,16]. The second way of anticancer effect of Se is the inhibitory effect on the process of cancer cell proliferation by affecting the expression of the tumor suppressor gene (p53 gene) and the apoptosis suppressor gene (Bcl-2 gene) [16].

Malignant tumors have become the leading cause of death worldwide, and despite available treatment options such as surgery, chemotherapy, immunotherapy, and radiotherapy, which are effective, they are also burdened with side effects and the development of drug resistance by malignant tumor cells [17]. Nano-Se is the Se with the lowest toxicity; it is usually zero-valent and can be absorbed directly by humans. It also has other properties—it dissolves in water and shows greater bioactivity than Se [18]. The anticancer activity of synthesized Se-containing compounds was assessed—PVP (polyvinylpyrrolidone). SeNPs have high bioavailability and low toxicity, and retain high anticancer activity. Studies have shown that PVP SeNPs demonstrate anticancer efficacy, which significantly increases in combination with RGD peptide, transferrin or folic acid [19,20]. Additionally, it was noted that PVP SeNPs may influence the effectiveness of drugs used and increase ROS production during radiotherapy [21]. Due to the use of polymers, Se nanoparticles achieve a stronger anti-cancer effect, acting selectively on cancer cells and reducing side effects. Additionally, polymers increase the stability of Se nanoparticles during their preparation, storage, and use [22].

## 2. Se and Malignant Tumors

More and more research confirms the influence of Se on the occurrence of malignant tumors. A large proportion of cancer cells are selenophilic, but selenide, which mediates the synthesis of Sec, has a toxic effect. In cancer cells, selenide must be detoxified through the synthesis of selenophosphate 2 (SEPHS2). This process takes place in cancer cells, i.e., when Se supplementation exceeds a certain dose, the growth of cancer cells is impaired [23]. Observational studies have shown that people with adequate levels of Se in their diet and, therefore, in their tissues, have a lower risk of developing cancer, and selenium levels in blood plasma may decline before some cancers develop [24]. A clinical study in 1973 compared Se values in samples from 111 patients who would later develop cancer with 210 cancer-free patients. The study results indicated that the highest quintile of Se in blood serum was half that of study participants from the lowest quintile [25]. In turn, other studies have shown that an increased supply of Se does not reduce the incidence of prostate cancer, lung cancer, colorectal cancer, and all other primary cancers, but the study was conducted in the USA and Puerto Rico where there is no Se deficiency compared to Europe; therefore, Se supplementation was not necessary here [26]. It can therefore be speculated that Se may influence the incidence of malignant tumors if its supplementation is properly balanced. An interventional study was conducted on the general population, which was divided into groups—one group received salt enriched with Se, and in this study group the incidence of primary liver cancer was 35% lower than in the study group that did not receive salt supplementation enriched with Se. When supplementation with selenium-enriched salt was discontinued, the incidence of primary liver cancer began to increase [27]. Se has also been proven to protect against breast cancer, which has the highest incidence of any cancer in the world [28]. Randomized, double-blind studies analyzed male patients diagnosed with basal cell, whose daily intake of Se was supplemented with 200 μg of Se or placebo for a period of 4.5 years. After 6.5 years, a significant reduction of as much as 63% in the secondary endpoint, i.e., the incidence of prostate cancer, was observed in men whose diet included 200 μg of Se [29]. A similar analysis showed that selenium is inversely associated with adenoma and malignant tumor of the large intestine [30]. Such test results are influenced by the stage and type of cancer as well as the chemical form and bioavailability of Se. Due to the fact that related studies often show contradictory results and conclusions, attention should be paid to the need to conduct further scientific research.

## 3. Pro-Oxidant Effect of Se

**Figure 1:**
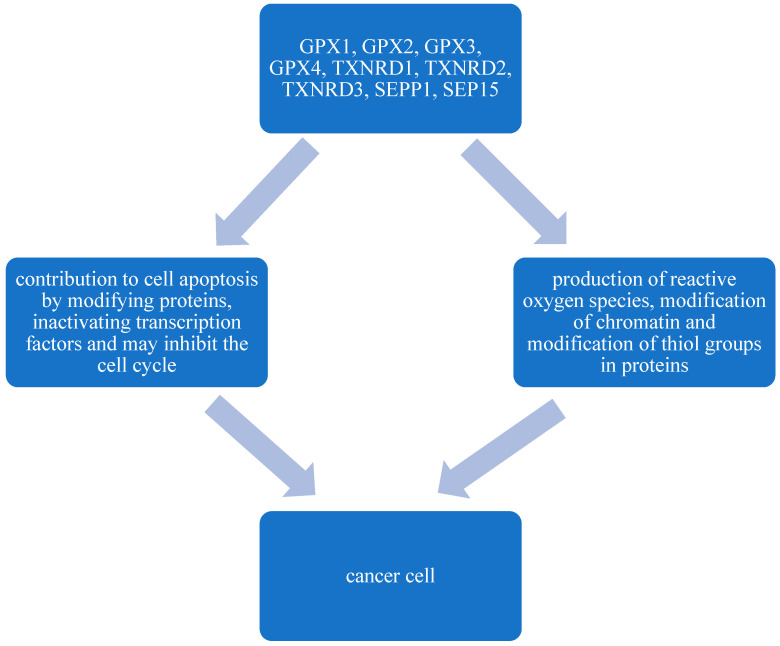
Se and cancer cells [30].

It has been shown that the strong pro-oxidant effect of Se disrupts the pro- and antioxidant balance by creating many reactive oxygen species. The effect of Se on cancer cells includes the production of reactive oxygen species, modification of chromatin, and modification of thiol groups in proteins. It has been shown that the element may also contribute to cell apoptosis by modifying proteins and inactivating transcription factors, and may inhibit the cell cycle (Figure 1) [30]. It is suggested, based on existing research, that Se may be helpful in the treatment of established cancers by cytotoxically damaging cancer cells. It has also been shown that SeO_2_ is used in the treatment of various malignant tumors, including lungs, the uterus, and prostate. It is also interesting that the use of Se can enhance the effect of radiation on hormone-independent, well-developed prostate tumors [31].

Studies conducted by Muecke R. et al. [32] showed that Se supplementation offers radioprotective potential in cancer patients and is promising as an adjuvant treatment option in patients with relative Se deficiency [32]. On the other hand, regarding Se supplementation Jiang J. et al. [33] in their study noticed that although body Se levels are associated with the occurrence and progression of prostate cancer, conventional Se supplementation does not appear to have the expected effect on prostate cancer in the general population [33]. Furthermore, the retrospective studies conducted by Pfister C. et al. [34] showed that Se deficiency is common in cancer patients admitted to an oncology rehabilitation clinic. Se supplementation during rehabilitation effectively corrected Se deficiency in most cases. The positive effects of rehabilitation lasted longer if the Se level did not decrease after rehabilitation [34].

Regarding malignancy, hydrogen Se (metabolite of inorganic Se) takes part in inducing apoptosis and ferroptosis in tumor cells. Therefore, a sufficient level of Se in the blood is crucial for supporting cellular function; also, it is possible that inorganic Se may obtain a value in anticancer therapies [35].

## 4. Average Se Level in the Blood, Serum, and Cancer

Se in the form of organic, inorganic compounds and nanoparticles has anticancer effects, but the effectiveness of this action depends on many factors, including the chemical form of Se, dose, type of cancer cells, and the stage of disease advancement [36].

It was shown that when the average Se level in the blood serum was more than 350 μg/L, no cancers of the colon, rectum, pancreas, kidneys, breasts, ovaries, lungs, and prostate were detected in this population [32]. The average selenium level in blood serum in patients diagnosed with cancer was below 100 μg/L [Table 1].

Studies have confirmed that in people with a serum Se concentration of approximately 120 μg/L, there is a several-fold reduction in the overall number of cancer cases and disease-related mortality [37]. Retrospective association studies have shown that in the vast majority of cases, there was a several-fold increase in the risk of developing lung or laryngeal cancer in people with low Se levels in the blood. Prospective association studies (measuring the Se level in the blood several years before the onset of the disease) have proven an inverse correlation between the Se concentration and the risk of developing malignant lung cancer. Randomized studies with Se supplementation showed a nearly three-fold reduction in the risk of lung cancer, provided that the initial Se concentration did not exceed 106 μg/L in the blood serum [37,38,39]. Significantly increased Se content in the blood serum associated with its supplementation was associated with an up to 25% increase in the risk of lung cancer. An inverse correlation has been shown between Se intake and the risk of developing bladder cancer [40] and pancreatic cancer [41], a malignant ovarian tumor [42] and malignant prostate tumor [43,44]. Higher Se levels were also found in breast cancer tissues than in surrounding cells [45], while other scientific studies did not confirm the inverse relationship between the concentration of Se in the blood and the risk of breast cancer [46]. The influence of Se(IV) oxide has been proven (SeO_2_) on the expression of p53 and Bcl-2 genes in three tested lung cell lines: PG, A549 and GLC-82 [14,16,47], along with increasing the concentration SeO_2_ decreased the Bcl-2 concentration; however, this was only in the case of A549 cells. p53 protein levels increased in all three cell lines (PG, A549, and GLC-82). The effect of Se on induction in lung cancer cell lines (U-1285 sensitive to doxorubicin; U-1285-dox insensitive to cytostatics) was also determined [48,49]. It has been proven that Se at low concentrations under physiological conditions has a protective function against gene- and cytotoxicity caused by oxidative stress, and also stimulates DNA repair [50,51]. It has been discovered that Se in low doses acts as an antioxidant in healthy and cancer-affected cells, and therefore cancer cells are protected against the effects of DNA damage accumulating in the initial phase of tumor development [52]. It has been shown that Se supplementation may contribute to reducing the risk of DNA mutations and carcinogenesis by limiting the incidence of DNA adducts and chromosomal breaks. It was assumed that the influence of Se on the risk of developing malignant tumors and their course may depend, in addition to the Se concentration, on the inherited molecular background. Genes encoding selenoproteins include: GPX1, GPX2, GPX3, GPX4, TXNRD1, TXNRD2, TXNRD3, SEPP1, and SEP15 [53].

**Table 1:** Average selenium level in blood serum in patients diagnosed with selected malignant tumors [54,55,56,57,58,59].

Type of Cancer	Average Selenium Level in Blood Serum	References
Papillary Thyroid Cancer (PTC)	79.15 μg/L	[54]
Laryngeal Cancer	66.8 μg/L	[55]
Breast Cancer	64.4 µg/L	[56]
Lung Cancer (Stage I)	69 μg/L	[57]
Malignant Melanoma	85.15 μg/L	[58]
Colorectal Cancer	100 μg/L	[59]

## 5. The Form of the Element and Biological Functions

Se occurs in the form of inorganic and organic compounds [60]. A total of 25 selenoproteins have been detected in humans [35]. The element supplied through food or dietary supplements occurs in organic (selenomethionine, selenocysteine) and inorganic (selenites, selenates) forms. Despite the different course of transformation of the forms of this element from organic and inorganic compounds, the final product is selenide hydrides, which are a direct Se donor during the synthesis of selenoproteins [61]. Se has an antioxidant effect and affects tissues and cells by removing reactive oxygen species (ROS), especially eliminating the effect of hydroperoxides in organs such as the heart, liver, kidneys, thyroid, and brain [62,63,64]. The biological functions of Se result from the presence of selenocysteine amino acids in proteins. These include thioredoxin reductases, selenoproteins, glutathione peroxidases, and iodothyronine deiodinases [15].

### Chemopreventive Effect of Selenium Supplementation

According to the recommendations of medical societies, Se should be supplemented. The basis for this recommendation is the result of research on Se consumption, which showed a deficiency of this element in the populations of many European countries. The correct supply of Se is one that ensures optimal activity of selenoprotein P, which is a Se transporter from the liver to tissues [65]. It has been shown that both deficiency and excess may have an adverse effect on the functioning of the body, thus increasing the risk of hypertension, thyroid dysfunction, limitations of the immune system and malignant tumors [66]. Due to the diverse occurrence of Se in particular regions of the world, Se supply standards differ on individual continents and even in individual countries. The normal concentration of Se in the blood is considered to be 70–150 μg/L [67]. Research shows that Se supplementation has a positive effect on the immune response to various harmful conditions occurring in the human body. Supplementation affects innate and acquired immunity [46,68]. Based on previous research, selenium supplementation has an immunomodulatory effect because it regulates immune cells [69,70].

Se, an essential mineral, is obtained from food products, and its total amount in humans ranges from 3 to 20 mg [71]. The main sources of selenium in the human diet are cereals, meat, fish, and eggs. The concentration of selenium varies depending on the type of food and its origin. Cereals are the main source of this element, but the Se content in cereals is low and ranges from 0.01 to 0.55 μg/g. Brazil nuts contain the most Se—512 g/g [72]. The main source of Se in plants is the soil (Se value above 0.4 mg/kg—soil rich in Se, and below 0.1 mg/kg—soil poor in Se) [73]. The World Health Organization (WHO) recommends an average selenium intake of 55 μg/day for adults, and the upper acceptable limit for Se intake is 400 μg/day.

For many years, Se has been studied in terms of the effectiveness of the treatment of lung cancer in terms of its chemo preventive effect [74]. It has been shown that Se supplementation can reduce the incidence of lung cancer in people with low baseline blood Se levels. The role of Se in preventing oxidative DNA damage and its impact on DNA repair, which plays a role in the process of carcinogenesis, has also been confirmed [75]. The influence of Se on the incidence of malignant tumors has been proven, but the use of supplementation with inorganic Se (Inorg-Se) and organic selenium (Org-Se) is limited by poor absorption and increased toxicity. Achieving an Se concentration of 106 ng/mL in blood serum reduced the risk of lung cancer, while a concentration of 121.6 ng/mL increased the risk of lung cancer [76]. Exposure to selenomethioine (Se Met) has also been shown to increase radiosensitivity in human lung cancer cell lines without damaging adjacent healthy tissues. It should be taken into account that the doses that have been used in cell studies may not be sufficient; so, increasing the doses for in vivo studies should be considered, and, therefore, their appropriate dose should be determined [77].

## 6. Selenium Nanoparticles

Nanotechnology combines fundamental multidisciplinary research with fundamental applications to create structures and materials ranging in size from 0.1 nm to 100 nm. In medicine, the field of nanomedicine has been created related to nanotechnology. Nanomedicine is a field of science and technology that, thanks to the combination of molecular instruments with knowledge about living organisms, can be used to identify, treat, and prevent diseases and injuries, while reducing pain and protecting and improving human health [78]. Thanks to these possibilities, nanomedicine has become very helpful in both the diagnosis and treatment of cancer. Due to its anticancer properties, Se is used in nanotechnology as Se nanoparticles (Se NP). Lai et al. [79] developed a ruthenium complex containing selenium with natural killer (NK) cells, enhancing immunotherapy against prostate cancer with this combination [79]. Liao et al. [80] investigated the effect of selenium nanoparticles on cancer treatment by increasing the level of mir-16 [80]. Se nanoparticles are also found in dietary supplements and therapeutic agents; their action has no side effects, even in the treatment of lung cancer. The impact of Se nanoparticles in reducing the effect of oxidative stress on cells has also been documented [81].

### Nanoselenium in Medicine

Studies have proven that Se NP have a beneficial effect in the chemoprevention of lung cancer, providing a potentially new drug with anticancer activity and as a carrier of anticancer drugs [82]. Tian et al. [83] proved in their research that nanoselenium in combination with radiofrequency therapy significantly inhibits the expression of proteins related to the proliferation of CCND1 and c-Myc and proteins related to the intermingling of MMP2 and MMP9, thus affecting lung cancer cells and causing their apoptosis [83,84]. In turn, ebselen—i.e., a synthetic organoselenoid molecule with anti-inflammatory, antioxidant and cytoprotective properties—has anti-cancer properties by inhibiting the activity of thioredoxin in tumor cells, inducing the apoptosis of tumor cells [85]. Compared to organic selenium and inorganic Se, Se nanoparticles have lower cytotoxicity and show higher anticancer effectiveness [86]. Gao et al. [86] showed that the combination of a selenium nanoparticle with irinotecan increases cytotoxicity towards HCT-8 cancer cells, increases the level of p53 expression, and increases the sensitivity of HCT-8 cell DNA to the induction of apoptosis [86,87,88]. Liao et al. [80] demonstrated that Se NP exhibited anticancer effects on prostate cancer cells by increasing the expression of miR-16 [80]. Additionally, Se NP has been shown to inhibit tumor cell proliferation by using them as drug carriers to regulate protein and DNA biosynthesis and protein kinase C activity [89,90]. However, it should be remembered that Se NP easily aggregate in water and have low dispersibility, causing a decrease in the level of bioactivity and bioavailability [91]. However, Se NP modified with polysaccharides and proteins show high bioactivity, stability, and low toxicity [92]. Jin et al. [93] investigated the embedding of Se NP in a triple β-helix, demonstrating that D-glucan inhibited the proliferation of acute myeloid leukemia cells [93].

Currently, work is being done on the use of increasingly broader groups of nanomedicines in the clinical treatment of lung cancer, such drugs include polymeric micellar paclitaxel [94], CRLX-101 [95,96] and other drugs that are currently in the clinical trial phase. Unfortunately, Se medicine is still rarely used. This is mainly due to the search for more efficient nanomaterials with low toxicity, a simple structure, and high biological stability [88]. An additional direction hindering the introduction of Se nanomedicine are difficulties in the path from activities in the laboratory to introduction to the industrial market. Problems related to parameter optimization and methodological problems also pose an obstacle, so it is crucial to design ideal sizes of selenium nanoparticles in the early phase of their creation. The clinical use of Se nanomedicine in the treatment of lung cancer requires work on a more effective Se NP based on the achievements of industrial production and the clinical needs of the patient.

**Figure 2:**
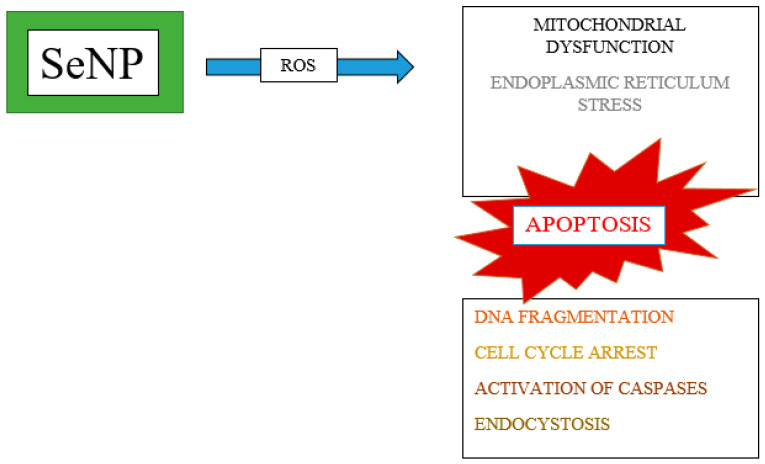
The effect of nanoselenium on a cancer cell [97].

Due to reduced toxicity and targeting, Se nanoparticles are more effective in the treatment of malignant tumors than other Se compounds. The uptake of nanoparticles by malignant cells occurs through the process of endocytosis. Nanoparticles act as pro-oxidants, increasing the formation of ROS, resulting in endoplasmic reticulum stress, mitochondrial membrane fission, apoptosis, DNA fragmentation, and cell cycle arrest (Figure 2) [97].

Se nanoparticles are used in the treatment of various disorders related to inflammation and oxidative stress, including malignant tumors, diabetes, arthritis, liver fibrosis, drug toxicity, and nephropathy [97]. Thanks to research conducted by Tan et al. [97] it has been proven that various Se nanoparticles can be used not only in the treatment of malignant tumors through chemotherapy but also in cancer diagnostics [97].

## 7. Summary

Se influences cell growth and development by maintaining the redox balance. The role of Se in cell death is not fully explained, but its involvement is in various types of cell death: apoptosis, ferroptosis, autophagy, and pyroptosis. Se regulates many ways of cell death using different cellular pathways. Se, as a microelement necessary for the proper functioning of the human body, is also used in the prevention and treatment of cancer. Se in the form of organic, inorganic compounds and nanoparticles has anticancer effects, but the effectiveness of this action depends on many factors, including the chemical form of selenium, dose, type of cancer cells, and the stage of disease advancement. The influence of Se on the incidence of malignant tumors has been proven, but the use of supplementation with inorganic Se (Inorg-Se) and organic Se (Org-Se) is limited by poor absorption and increased toxicity. Se is obtained from food products, and its total amount in humans ranges from 3 to 20 mg. Scientific research has shown that Se may be effective in supporting the treatment of malignant tumors, which continue to pose a challenge to medicine. The interesting results of previous research constitute the basis for further analysis regarding the anticancer effect of Se. Nevertheless, due to reduced toxicity and targeting, selenium nanoparticles are more effective in the treatment of malignant tumors than other selenium compounds.

## Data Availability

Not applicable.
